# Exposure of the Main Italian River Basin to Pharmaceuticals

**DOI:** 10.1155/2011/989270

**Published:** 2011-09-19

**Authors:** Federico Ferrari, Agata Gallipoli, Matteo Balderacchi, Maria M. Ulaszewska, Ettore Capri, Marco Trevisan

**Affiliations:** ^1^Istituto di Chimica Agraria ed Ambientale, Università Cattolica del Sacro Cuore, 29122 Piacenza, Italy; ^2^AEIFORIA Spin-Off Company dell' Università Cattolica del Sacro Cuore, 43036 Fidenza, Italy

## Abstract

This study give a preliminary survey of pharmaceutical contamination and accumulation in surface waters and sediments along the river Po basin (74,000 km^2^, the largest in Italy), a strategic region for the Italian economy: it collects sewage from a vast industrialized area of Italy (Autorità di Baciono del fiume Po, 2006, 2009). 10 pharmaceuticals (atenolol, propanolol, metoprolol, nimesulide, furosemide, carbamazepine, ranitidine, metronidazole, paracetamol, and atorvastatin) from several therapeutic classes were searched in 54 sampling points along the river Po from the source to the delta, and at the mouth of its major effluents. In water samples were found pharmaceuticals in the range of 0.38–0.001 **μ**g/L, except for furosemide (max conc. 0.605 **μ**g/L), paracetamol (max conc. 3.59 **μ**g/L), metoprolol (never detected) and for atenolol (not analysed). In sediment samples, only paracetamol was not detected, while the others were generally found in the range of 0.4–0.02 **μ**g/kg *ww* with high concentrations for atenolol (max conc. 284 **μ**g/kg *ww*) and furosemide (max conc. 98.4 **μ**g/kg *ww*). The findings confirm also STPs as point sources of contamination. Despite of the much evidence for the adverse effects of pharmaceuticals in the aquatic environment, the observed low levels cannot be considered to pose a serious risk to human health; further studies are necessary for a comprehensive risk assessment.

## 1. Introduction

Today, one of the most relevant environmental issues is the occurrence of pharmaceutically active substances in surface waters, wastewater effluents, and also sediments. An important entry route of pharmaceuticals into the environment is via conventional sewage-treatment plants (STPs). It has been extensively shown [1–7] that STPs are unable to completely remove contamination by pharmaceuticals. For the majority of drugs, removal by conventional biological treatments seems inefficient, such that contamination remains in the water effluents [8–10]. A recent study performed by Zuccato's research group showed that the total removal rate in STPs was generally lower than 40% (http://www.cwwt.unsw.edu.au/ywp2006/papers/YWP%20P.3.pdf “Behavior of pharmaceuticals in sewage treatment plants”). 

Pharmaceuticals, because of their continuous use and entry into the environment, are considered as “pseudopersistent compounds” [11]. They are introduced into the environment into water and/or sewage through manufacturing processes, improper disposal, and metabolic excreta, in the form of parent compounds or as metabolites. In addition, drug residues have been found in the terrestrial environment; pharmaceuticals with acidic properties and high log *K*_ow_, mainly antibiotics, show affinity to soil, sediment, and sludge [12–14], in contrary to some drugs degradation that can be promoted by microbial activity present in riverine or lagoon sediments [14, 15], while their transport could be mediated by colloids present in riverine water [16]. In this case, the disposal of biosolids from STPs and animal wastes, which are applied to land, represents the major inputs into the environment [17, 18]. 

Even if pharmaceuticals are only detected in water at trace levels such as ng/L to low *μ*g/L [19–22], these concentrations may be of concern because these compounds are developed to be biologically active [23]. Despite this increasing concern about the possible impact of human pharmaceuticals on the environment, there are no regulations in European law which set threshold values for drug residues in the environment. 

The European Union has established a community framework for water protection and management, with the Directive 2000/60/EC of the European Parliament and the Council of 23rd October 2000. This Framework Directive provides for the management of groundwater, inland surface waters, and transitional and coastal waters in order to prevent pollution, to promote sustainable water use, to protect the aquatic ecosystem, and to improve the status of the aquatic environment. The European Union (EU) has also designed a document in which a stepwise procedure for the Environmental Risk Assessment (ERA) of pharmaceuticals is established. This document has been revised several times and was finally accepted and written as the guideline into European law in June 2006 (guideline on the environmental risk assessment of medicinal products for human use).

This guideline requires that “an application for the marketing authorisation for a medicinal product for human use shall be accompanied by an environmental risk assessment,” in order to assess “those risks to the environment arising from use, storage, and disposal of the medicinal product.” The proposed ERA in this document is a two-tiered analysis of potential environmental risk. Phase I should consist of an estimate of the exposure of the environment to a drug, taking into account the estimated yearly production, the market penetration, and the predicted drug degradation in STPs and its fate into the environment. Phase II (divided into tier A and tier B) should establish drug physicochemical, toxicological, and pharmacological properties. Phase II testing is required for all substances with a predicted environmental concentration (PEC) of 0.01 *μ*g/L or higher and/or with a specific mode of action such as a direct or indirect interaction with a receptor [24, 25]. 

Indeed the regulation has been endorsed water because contamination occurred in different countries. The presence of pharmaceuticals in surface waters has been assessed in the river Po basin by means of a voluntary initiative coordinated by the Italian Civil Protection with our institute during the years 2006 and 2007. The monitoring programme was carried out in the river Po basin from Pian del Re (source) to Porto Tolle (delta).

## 2. Experimental

### 2.1. Chemicals and Materials

Based on *previously mentioned investigations* about the water quality of some rivers and STP outlets in northern Italy measuring concentrations of various pharmaceuticals, this research focused on the following chemicals detected at least once previously: atenolol, propanolol, metoprolol, nimesulide, furosemide, carbamazepine, ranitidine, metronidazole, paracetamol, and atorvastatin. The individual standard solutions of pharmaceuticals were prepared in pure methanol and stored at −20°C. Methanol and acetonitrile (chromatography grade) were purchased from *Merck* (Italy). The active pharmaceutical ingredients were obtained from commercial products at the concentrations certified on the label.

### 2.2. Sampling and Sample Preparation

**Figure 1:**
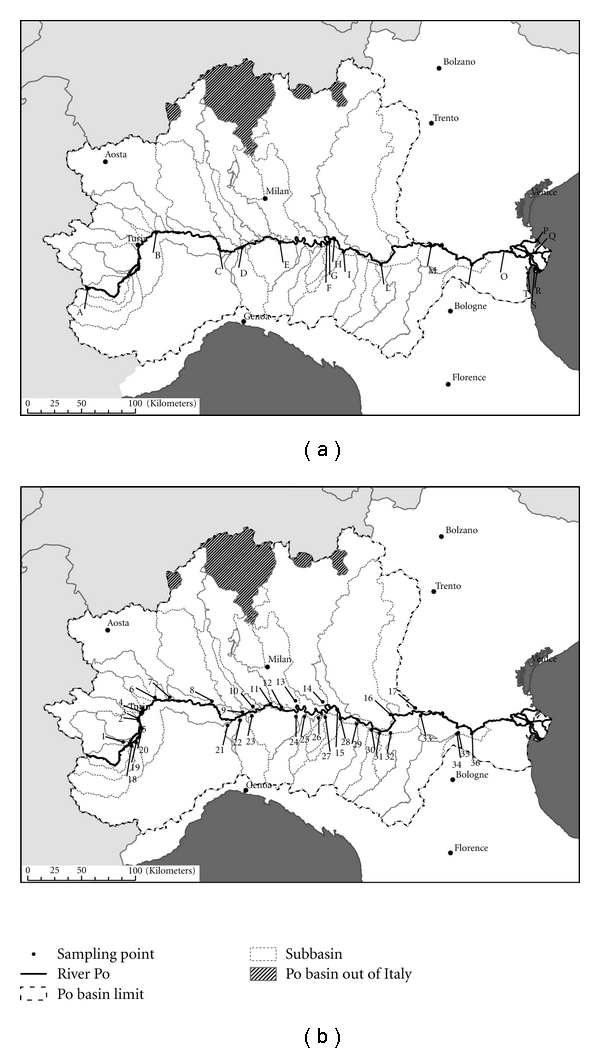
Sampling points along river Po (a) and its tributaries (b).

Aqueous and sediment samples were collected from each sampling point, *in means of three subsamples for each point. *Eighteen sampling points were identified along the river Po starting from its source and then downstream of the major urban areas or immediately downstream of the confluence of the effluents (Figure 1(a)). Particularly relevant because of their high flow rate or human density within their subbasins, 36 sampling points were identified at the mouth of all the major Po effluents: within these, one was placed at the exit of an urban STP (town of Cremona), and one was placed at the confluence with an artificial channel (Cavo Napoleonico) connecting two hydrographical basins (Po and Reno basins) (Figure 1(b)).

Both water and sediment samples were collected manually in the centre of the stream section, sedimented by a Van Veen sampler [26] or by hand/spade sampling where the water depth was not too deep, and stored in polypropylene bags, while water samples were collected in darkened-glass bottles. All the samples were immediately refrigerated at 4°C during the transport to the laboratory and until extraction and analysis. 

Aqueous samples were extracted by solid phase extraction (SPE) cartridges and analysed by reversed-phase liquid chromatography/mass spectrometry (HPLC-MS) [27]. Two different extractions by means of SPE cartridges were chosen to find a multiresidue method of extraction for pharmaceuticals from a wide spectrum of chemical classes. Bond Elut PPL (Superchrom, Italy) is designed for highly polar species and for large-volume water samples (particle size of 125 *μ*m, pore size of 150 Å, and surface area of 600 m^2^/g, functional group: SDB—base deactivated silica). The discovery DSC-18 SPE tubes (Supelco, Italy), designed for less polar chemicals, had a silica gel and polymerically bonded octadecyl with high carbon loading (18% C) sorbent matrix, with particle size of 50 *μ*m, pore size of 70 Å, and a surface area of 480 m^2^/g.

For the extraction, the PPL Bond Elut 3 mL tubes and Discovery DCS-18 6-mL tubes (500 mg) were activated and conditioned with methanol (6 mL) and then ultrapure water (12 mL) with application of mild suction by a vacuum manifold. Water samples (1000 mL) were passed through the cartridges at a flow rate of 5 mL/min. Sorbed analytes were then eluted with acetonitrile (10 mL) into a 10 mL glass test tube. The solvent was evaporated under a nitrogen stream and redissolved in methanol (1 mL), which solution was *then* transferred to an autosampler vial for HPLC-MS analysis. 

The extraction from sediments (50 g ww) was performed via soxhlet apparatus by means of acetone as solvent (200 mL) followed by dehydration on sodium anhydrous sulphate and reduction to small volume (1 mL) by means of Rotavapor an nitrogen flux. The extract was then analysed following the same procedure used to analyse water sample extract.

### 2.3. HPLC/MS Determination

HPLC-MS analysis was performed by a Thermo Electronic Corporate HPLC-MS with a Surveyor MSQ Plus Finnigan single-quadrupole mass detector operated in electrospray ionization (ESI) mode. The HPLC was a Thermo quaternary pump with a degasser and autosampler. The HPLC separation was performed on a Phenomenex column, Synergi 4 *μ*m Hydro-RP 80(A) 150 mm × 4.60 mm id. The mobile phase was acetonitrile and water with 0.1% of formic acid at a constant flow rate of 0.3 mL/min. The analyses were done in ESI negative for furosemide, nimesulide, and atorvastatin and in ESI positive for the other compounds. For both methods, the gradient of separation was 65 : 35% of water : acetonitrile from 0 min to 7 min, increasing to 80% of acetonitrile over 25 min, static at 80% of acetonitrile for 7 min, decreasing to 30% of acetonitrile over 3 min, and static at 30 : 70% of acetonitrile : water for 3 min (38 min total time). The MS detector probe was set at 600°C and the needle at 4 kV for the ESI positive method, whereas the probe was set at 570°C for the ESI negative method. The detection of all pharmaceuticals was performed in SIM for the quantification and full scan for the identification. Data were acquired from m/z 200 to 500. The software used for control, analysis, and quantification was Xcalibur 1.4. 

Linearity was tested assessing signal responses of analytes in standard solutions and in matrix extracts over a range of concentrations from 0.001 up to 1.0 mg/L (mg/kg). Analytical signal of standard solution was compared with the signal of a blank water extract spiked after extraction with target compounds. Recoveries from water and sediment ranged from 65% to 80% for of the pharmaceuticals at the two concentrations of 0.1 and 1.0 mg/L (mg/kg). Recovery for atenolol was assessed only in sediment within the range described above.

### 2.4. CEC and OC Determinations

Cationic exchange capability was assessed according to the Barium chloride and triethanolamine method [28], and organic carbon content was assessed by ISO 14235 [29], Walkley-Black method.

## 3. Results and Discussion

This work shows the results of a monitoring project of 10 pharmaceuticals in water and sediment collected in 2006 and 2007 in the basin of the Po river and all its effluents. The drugs for analysis were chosen so as to have a variety of pharmaceuticals representative of prescription and nonprescription classes, belonging in particular to seven therapeutic classes: *β*-blockers, anti-inflammatories, ulcer healers, diuretics, lipid-regulator agents, antiepileptics, and antibiotics (Table 1). Drugs have been investigated to understand if they might be accumulated in the aquatic environment and pose a risk to living organisms. Most of the previous research projects carried out in the area by Zuccato's research group focus only on pharmaceutical residues in water samples, ground waters, or STP effluents; therefore, our choice of considering also sediment samples was done to better understand the degree of accumulation resulting from different sources and trends. Due to limitations of resources, the compounds investigated were the most used and the most frequently detected in other European surveys. In the first year, preliminary screening was done on five pharmaceuticals, whereas in the second year, the study was extended to 10 pharmaceuticals. As reported in Table 1, these pharmaceuticals span a wide range of physicochemical properties.

**Figure 2:**
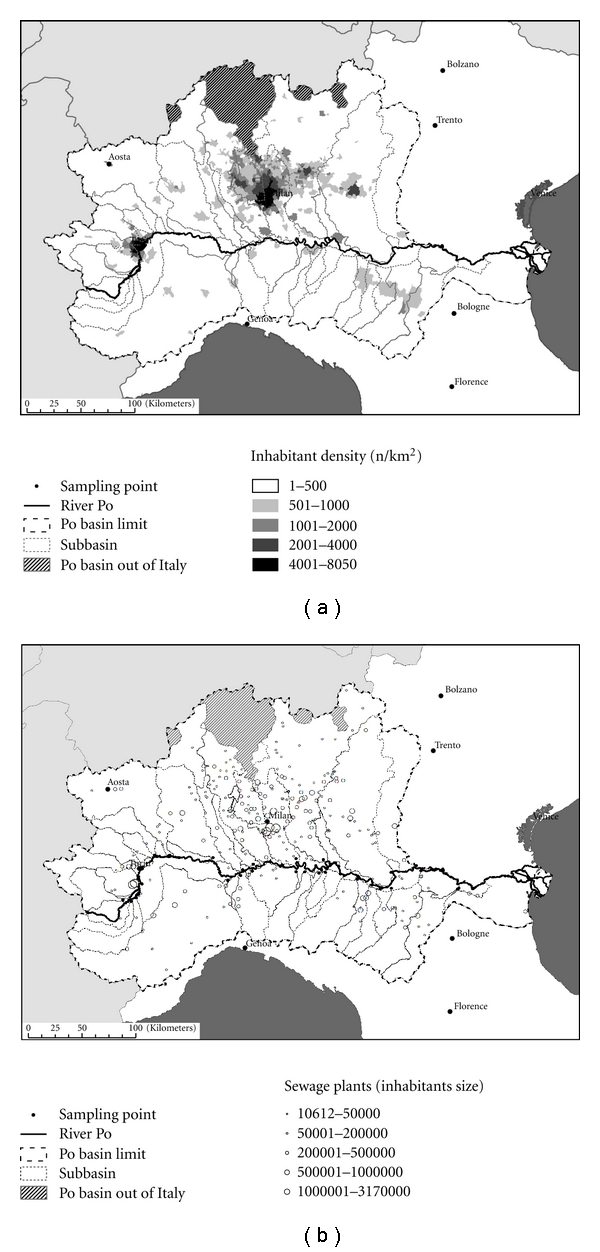
Inhabitant distribution (a) and sewage plants distribution (b) in river Po basin and its subbasins.

The Po river basin is the largest and the most important in Italy, covering an area of 74,000 km^2^ [30, 31]. The Po area is a strategic region for the Italian economy, with significant agriculture, livestock, industry, and tourism, and it collects sewage from a vast industrialized area of northern Italy that represents an intense and continuous loading into the STP system (Figure 2) and subsequent emission. Therefore, it may be considered the worst realistic and representative Italian scenario to estimate the level of contamination in surface-water bodies. 

Table 2 indicates the pharmaceuticals concentration found in water and sediments, including the cationic exchange capability and the organic carbon content. Amongst the 10 pharmaceuticals of interest, two (atenolol and metoprolol) were not detected in the water samples. Most pharmaceuticals were found in the range of 0.38–0.001 *μ*g/L, except for furosemide (max conc. 0.605 *μ*g/L) and paracetamol which was detected at higher concentrations in almost all samples (max conc. 3.59 *μ*g/L) (Table 2). 

In sediment samples, only paracetamol was not detected, while the others were generally found in the range of 0.4–0.02 *μ*g/kg. High concentrations were found in sediment samples for atenolol (max conc. 284 *μ*g/kg) and furosemide (max conc. 98.4 *μ*g/kg). 

These results might indicate a correlation between concentration and the physicochemical properties of the drugs, in particular their log *K*_ow_. Chemical compounds with higher *K*_ow_ show greater affinity to hydrophobic matrices rather than to water. So, it might be predicted that pharmaceuticals such as atorvastatin, nimesulide, furosemide, and ranitidine are more concentrated in sediment samples, whereas our investigation indicates atenolol (log *K*_ow_: 0.5) as the principal drug residue in sediments. In addition, paracetamol, which has a similar log *K*_ow_ (0.46), was never detected in sediment samples. On the basis of the observed results, plotting maximum concentration (*C*_max._) of pharmaceuticals versus log *K*_ow_ showed no relationship between concentration and log *K*_ow_. This is in agreement with the findings in [32, 33] and may be due to the historical accumulation of pharmaceuticals in the sediment as well as the characteristics of the sources of the contamination. STP inlet contaminates water discontinuously in time, and space while in parallel sediment burrow along the river can produce heterogeneous contamination of the sediment. 

All the sediments showed concentrations of most pharmaceuticals higher than 0.01 *μ*g/kg (except paracetamol), and in water samples in most cases, with the exceptions of atenolol and metoprolol, the concentrations found exceeded the threshold value set by European Medicines Agency (EMEA) [24] for water (0.01 *μ*g/kg). Such concentrations appeared to be in both the matrices, being correlated with the population living around each sampling point and with the characteristics of the STPs. In sediments from the Po effluents, atenolol was found at high concentration (108 *μ*g/kg) corresponding to the confluence with the Orco river, around which there is a population of 69854 inhabitants, and nearby STPs have a total nominal load of *45.000 inhabitants*. A concentration of 61 *μ*g/kg of atenolol was detected in the Chisola river, probably because of nearby STPs with a big nominal load (*ca. 314500 inhabitants*) which release their effluents. Surprisingly, a low amount of atenolol of 30 *μ*g/kg was found in Adda basin, in spite of ca. 1.687.000 inhabitants living around. This can be explained considering the nearest sampling point along the Po river (Monticelli), where the highest atenolol concentration was detected (283 *μ*g/kg). These results confirm atenolol as a priority pollutant [34].

Atenolol was not found in all water samples. A similar result was recently obtained by Kuster et al. [35] in two pilot monitoring studies in the Llobregat river basin. For furosemide, the high concentration detected in sediments sampled at the confluence with the Sesia river (98.42 *μ*g/kg) could be strange compared with the concentration found at Monticelli d'Ongina (17.81 *μ*g/kg) considering only the populations living around (ca. 632100 for the first one and ca. 500000 for the second one); however, this can be explained because of the presence of an STP with a big nominal load (100000) at 20 km distance from the sampling point on the Sesia river.

**Figure 3:**
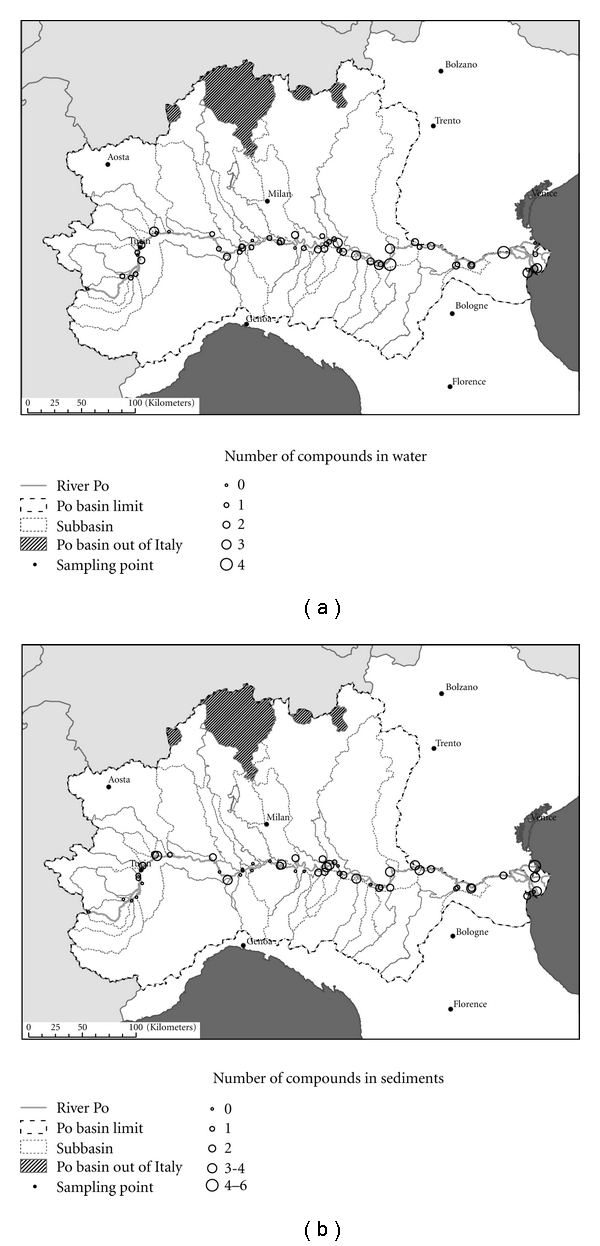
Number of compounds detected in water (a) and sediment (b) samples collected in river Po basin and its tributaries.

Despite the above two observations, it was quite difficult to observe a correlation between the concentration or the number of pharmaceuticals detected at each sampling point and the population and STP's nominal load around as shown in Figure 3 in which number of pharmaceuticals is reported per each sampling point along the Po river and its effluents, ordered by increasing population. A similar situation is observed if plotting number of drugs and sampling points ordered by increasing STP's nominal load.

To have an idea of the removal rate efficiency by STPs, waters sampled at Cremona city (sampling point H) can be considered. Their drug content is 0.079 *μ*g/L of paracetamol. On the other hand, waters coming out of Cremona STP (sampling point 15) contain carbamazepine, nimesulide and also a higher amount of paracetamol (0.486 *μ*g/L), indicating that STPs can sometimes act as sources of point contamination (Table 2). 

In Figure 3, the number of detected pharmaceuticals per sampling point along the Po river both in sediment and in water samples is reported. Considering water samples, the number of drugs generally increased in sampling points near the Po delta (from O to T), with a maximum value at the O site (Berra-Papozze). In contrast, it was not possible to observe any trend in the distribution of pharmaceuticals in water samples from the Po effluents.

## 4. Conclusions

This work presents the results of a voluntary monitoring project carried out in two consecutive years (2006-2007) in the most important Italian river basin with the aim to give a preliminary survey of the level of pharmaceutical contamination and accumulation in surface waters and sediments.

Of the 10 pharmaceuticals from several therapeutic classes, two (atenolol and metoprolol) were never detected in water samples; other drugs were at levels below 100 ng/L in most instances. This is in agreement with findings in water of previous investigations performed in small parts of the same region. The levels of pharmaceuticals detected in sediment samples were generally higher (in the range of *μ*g/kg), in particular for atenolol and furosemide.

Indeed no satisfactory correlation was observed between the environmental concentration and the distribution of pharmaceuticals and the resident population or the STPs' nominal loads around each sampling point; the findings confirm STPs as point sources of contamination and a discontinuous accumulation in the sediment. 

Despite much evidence for the adverse effects of pharmaceuticals in the aquatic environment, the observed low levels cannot be considered to pose a serious risk to human health; further studies are necessary for a comprehensive risk assessment because the resident population could be exposed by multiple sources such as irrigation, drinking, recreational, and food uses. In addition, the synergistic effects of a mixture of different compound classes are still unknown [11, 27, 36]. As noted, the Po river represents the most important Italian river basin because of its dimensions of the population living in and the economic activities performed in the basin. Water from the Po river is used for industrial purposes, in recreational facilities, in agriculture (for irrigation and cattle), in aquaculture plants, in particular in the area around Po delta, and almost along the entire basin surface, and ground waters represent an important source of drinking water (particularly around the cities of Ferrara and Rovigo) [37]. As an example, one of the biggest purification plants for drinking water is located at Pontelagoscuro (in our investigation sampling point N); in water sampled here, only one pharmaceutical was detected and that was at low concentration (carbamazepine 0.021 *μ*g/L). However, the level of pharmaceutical contamination is almost the same at Sacca di Goro (sampling point S), one of the most productive systems of clam farming in Italy, where also two other pharmaceuticals (atorvastatin and furosemide) were detected at levels of 0.023–0.027 *μ*g/L. A recent study, conducted at the river Po delta, [37] indicates that pharmaceuticals could be found in drinking water and how purification plants can be more efficient only if structured with new modules as granular-active carbon stage.

From these concerns is therefore foreseen a need to further assess if the pharmaceuticals detected in Po river waters would pose any risk for human health as well as for the terrestrial and aquatic organisms living in the basin.

## Figures and Tables

**Table 1:** Literature values (S. Castiglioni et al., 2004; R. Andreozzi et al., 2003; Banca dati Farmacoambiente; Drugbank) of the physicochemical properties and degradation behaviour of the selected pharmaceuticals.

Class of drugs	Pharmaceutical	Molecular weight (g/mol)	Log *K*_ow_	Stability in water	
*β*-Blocker	Atenolol	266.3	0.5	Stable for 40 d (5–25°C) *t*_50_45.2 h pH 7.4 (UV ray)	Moderate stability

Anti-inflammatory	Paracetamol	151.16	0.46 (experim.)
			0.27 (predicted)
	Nimesulide	308.3	2.56		
	Metoprolol	267	0.5		
	Propranolol	259	3.65		

Ulcer healing	Ranitidine	314.4	1.3 (experim.)	Stable 160 h pH 6.18,65°C	Prolonged stability
			0.79 (predicted)		
Diuretic	Furosemide	330.7	1.4 (experim.)	Stable 90 d pH 5.2	Prolonged stability
			2.71 (predicted)	Stable 96% 240 d pH 5.2	
Lipid regulator	Atorvastatin	558.6	5.7 (experim.)
			4.24 (predicted)
Antiepileptic	Carbamazepine	236	2.3 (experim.)	*t*_50_ 100 d	Prolonged stability
			2.10 (predicted)		
Antibacterial	Metronidazole	171	−0.1 (experim.)
			−0.15 (predicted)

**Table 2:** Concentration of 10 pharmaceutical compounds measured in water (*μ*g/L) and sediment (*μ*g/kg) samples collected from the Po river (from A to T), its effluents (from 1 to 36) and respective cationic exchange capability and organic carbon content. — = not analysed; n.d. = not detected, below limit of quantification.

Sampling site	Cationic	OC	Atorvastatin		Ranitidine		Propanolol		Carbamazepine		Atenolol		Metoprolol		Metronidazole		Paracetamol		Furosemide		Nimesulide	
	exch. cap.		sedim.	water	sedim.	water	sedim.	water	sedim.	water	sedim.	water	sedim.	water	sedim.	water	sedim.	water	sedim.	water	sedim.	water
												ng/g										
1	2.96	2.88	—	—	—	—	—	—	n.d.	n.d.	n.d.	—	—	—	—	—	n.d.	n.d.	n.d.	n.d.	n.d.	0.004
2	18.39	11.19	—	—	—	—	—	—	n.d.	n.d.	61.1	—	—	—	—	—	n.d.	n.d.	n.d.	n.d.	n.d.	n.d.
3	7.74	1.22	—	—	—	—	—	—	n.d.	0.005	n.d.	—	—	—	—	—	n.d.	n.d.	2.32	n.d.	n.d.	n.d.
4	8.45	6.08	—	—	—	—	—	—	n.d.	0.009	n.d.	—	—	—	—	—	n.d.	0.065	n.d.	n.d.	n.d.	n.d.
5	10.02	3.17	—	—	—	—	—	—	n.d.	n.d.	24.1	—	—	—	—	—	n.d.	n.d.	2.7	n.d.	n.d.	n.d.
6	11.2	1.23	—	—	—	—	—	—	n.d.	0.002	109	—	—	—	—	—	n.d.	0.666	2.36	n.d.	n.d.	0.085
7	12.3	14.59	—	—	—	—	—	—	n.d.	n.d.	n.d.	—	—	—	—	—	n.d.	n.d.	1.51	n.d.	n.d.	n.d.
8	11.72	5.91	—	—	—	—	—	—	n.d.	0.009	47.6	—	—	—	—	—	n.d.	n.d.	98.4	n.d.	n.d.	n.d.
9	13.89	1.75	—	—	—	—	—	—	n.d.	n.d.	n.d.	—	—	—	—	—	n.d.	0.297	n.d.	n.d.	n.d.	n.d.
10	20.29	14.1	—	—	—	—	—	—	n.d.	n.d.	n.d.	—	—	—	—	—	n.d.	n.d.	n.d.	n.d.	n.d.	n.d.
11	7.75	2.63	—	—	—	—	—	—	n.d.	n.d.	n.d.	—	—	—	—	—	n.d.	0.061	n.d.	n.d.	n.d.	n.d.
12	38.4	34.51	—	—	—	—	—	—	0.57	0.012	12.0	—	—	—	—	—	n.d.	0.055	0.559	n.d.	n.d.	n.d.
13	32.54	28.4	—	—	—	—	—	—	n.d.	0.063	12.7	—	—	—	—	—	n.d.	0.048	4.53	n.d.	n.d.	n.d.
14	8.75	1.75	—	—	—	—	—	—	n.d.	n.d.	30.7	—	—	—	—	—	n.d.	0.24	4.56	n.d.	n.d.	n.d.
15	10.49	9.56	—	n.d.	—	n.d.	—	n.d.	—	0.112	—	—	—	n.d.	—	n.d.	—	0.486	—	n.d.	—	0.15
16	14.35	12.8	0.220	0.028	n.d.	n.d.	0.069	0.007	n.d.	n.d.	0.33	—	n.d.	n.d.	n.d.	n.d.	n.d.	n.d.	0.627	n.d.	n.d.	0.001
17	39.58	61.73	n.d.	n.d.	n.d.	n.d.	0.158	n.d.	0.027	0.004	n.d.	—	n.d.	n.d.	n.d.	n.d.	n.d.	n.d.	0.397	0.605	n.d.	n.d.
18	27.31	25.24	—	—	—	—	—	—	n.d.	n.d.	n.d.	—	—	—	—	—	n.d.	0.176	n.d.	n.d.	n.d.	n.d.
19	9.4	1.41	—	—	—	—	—	—	n.d.	0.004	n.d.	—	—	—	—	—	n.d.	n.d.	n.d.	n.d.	n.d.	n.d.
20	9.21	2.09	—	—	—	—	—	—	n.d.	0.044	n.d.	—	—	—	—	—	n.d.	0.034	n.d.	n.d.	n.d.	n.d.
21	22.45	9.54	—	—	—	—	—	—	1.21	0.020	10.1	—	—	—	—	—	n.d.	0.527	8.63	n.d.	n.d.	n.d.
22	16.48	12.48	—	—	—	—	—	—	n.d.	n.d.	n.d.	—	—	—	—	—	n.d.	3.59	n.d.	n.d.	n.d.	n.d.
23	44.56	56.45	—	—	—	—	—	—	n.d.	0.115	n.d.	—	—	—	—	—	n.d.	n.d.	n.d.	n.d.	n.d.	n.d.
24			—	—	—	—	—	—	—	—	—	—	—	—	—	—	—	—	—	—	—	—
25	14.84	0.58	—	—	—	—	—	—	n.d.	n.d.	n.d.	—	—	—	—	—	n.d.	0.432	n.d.	n.d.	n.d.	n.d.
26	13.35	4.92	—	—	—	—	—	—	0.770	0.005	23.6	—	—	—	—	—	n.d.	0.227	n.d.	n.d.	n.d.	n.d.
27	20.28	10.7	—	—	—	—	—	—	1.26	0.003	16.3	—	—	—	—	—	n.d.	0.122	n.d.	n.d.	n.d.	n.d.
28	21.33	10.72	n.d.	n.d.	n.d.	n.d.	n.d.	n.d.	n.d.	0.004	2.93	—	n.d.	n.d.	n.d.	n.d.	n.d.	n.d.	n.d.	n.d.	n.d.	n.d.
29	27.23	9.93	0.288	n.d.	n.d.	n.d.	0.242	n.d.	0.040	0.112	n.d.	—	n.d.	n.d.	n.d.	n.d.	n.d.	0.486	n.d.	n.d.	n.d.	0.15
30	34.09	13.44	n.d.	0.128	n.d.	n.d.	n.d.	n.d.	n.d.	n.d.	n.d.	—	n.d.	n.d.	n.d.	n.d.	n.d.	n.d.	n.d.	0.048	n.d.	n.d.
31	21.83	10.1	n.d.	n.d.	n.d.	n.d.	0.094	n.d.	n.d.	0.014	n.d.	—	n.d.	n.d.	n.d.	0.023	n.d.	0.061	0.156	n.d.	n.d.	n.d.
32	34.76	21.67	n.d.	0.044	0.028	0.044	n.d.	n.d.	0.028	n.d.	n.d.	—	n.d.	n.d.	n.d.	0.068	n.d.	n.d.	n.d.	n.d.	n.d.	0.01
33	28.12	12.1	n.d.	n.d.	n.d.	n.d.	0.138	n.d.	n.d.	n.d.	0.156	—	n.d.	n.d.	0.16	n.d.	n.d.	n.d.	0.571	n.d.	n.d.	0.002
34	35.95	25.74	n.d.	0.016	n.d.	n.d.	0.42	n.d.	n.d.	0.004	n.d.	—	n.d.	n.d.	n.d.	n.d.	n.d.	n.d.	n.d.	n.d.	n.d.	n.d.
35	22.76	14.2	n.d.	n.d.	n.d.	n.d.	0.144	n.d.	n.d.	n.d.	n.d.	—	n.d.	n.d.	n.d.	n.d.	n.d.	0.09	n.d.	n.d.	n.d.	n.d.
36	30.59	22.5	n.d.	n.d.	n.d.	0.035	0.165	n.d.	n.d.	0.003	n.d.	—	0.425	n.d.	n.d.	n.d.	n.d.	n.d.	0.508	n.d.	n.d.	n.d.
A	18.96	14.55	—	—	—	—	—	—	n.d.	n.d.	n.d.	—	—	—	—	—	n.d.	n.d.	n.d.	n.d.	n.d.	n.d.
B	14.31	11.5	—	—	—	—	—	—	0.92	—	26.75	—	—	—	—	—	—	—	5.17	—	n.d.	—
C	1.15	10.43	—	—	—	—	—	—	n.d.	0.012	n.d.	—	—	—	—	—	n.d.	n.d.	n.d.	n.d.	n.d.	n.d.
D	6.42	0.19	—	—	—	—	—	—	n.d.	0.007	n.d.	—	—	—	—	—	n.d.	0.322	n.d.	n.d.	n.d.	n.d.
E	16.83	0.97	—	—	—	—	—	—	0.86	0.007	217.54	—	—	—	—	—	n.d.	n.d.	n.d.	n.d.	n.d.	n.d.
F	11.77	5.6	—	—	—	—	—	—	2.92	n.d.	15.29	—	—	—	—	—	n.d.	0.103	17.5	n.d.	n.d.	n.d.
G	35.33	20.56	—	—	—	—	—	—	1.86	n.d.	283.54	—	—	—	—	—	n.d.	0.373	8.66	n.d.	n.d.	n.d.
H	6.78	0.23	—	—	—	—	—	—	n.d.	n.d.	0.99	—	—	—	—	—	n.d.	0.079	n.d.	n.d.	n.d.	n.d.
I	8.37	3.84	0.16	0.035	n.d.	n.d.	n.d.	0.004	n.d.	n.d.	0.24	—	n.d.	n.d.	n.d.	n.d.	n.d.	n.d.	n.d.	n.d.	n.d.	n.d.
L	9.62	0.975	0.215	n.d.	n.d.	n.d.	n.d.	n.d.	n.d.	n.d.	n.d.	—	n.d.	n.d.	n.d.	n.d.	n.d.	n.d.	n.d.	n.d.	n.d.	0.001
M	21.07	11.68	n.d.	0.008	0.009	0.009	n.d.	n.d.	n.d.	n.d.	n.d.	—	n.d.	n.d.	n.d.	n.d.	n.d.	n.d.	0.728	n.d.	n.d.	n.d.
N	18.9	9.61	n.d.	n.d.	n.d.	n.d.	0.42	n.d.	n.d.	0.021	n.d.	—	n.d.	n.d.	n.d.	n.d.	n.d.	n.d.	0.728	n.d.	n.d.	n.d.
O	28.25	14.95	n.d.	n.d.	n.d.	n.d.	0.067	0.018	n.d.	n.d.	0.109	—	n.d.	n.d.	n.d.	0.013	n.d.	0.07	n.d.	n.d.	n.d.	0.002
P	23.21	20.88	n.d.	n.d.	0.036	n.d.	n.d.	n.d.	0.041	n.d.	0.304	—	0.256	n.d.	n.d.	n.d.	n.d.	n.d.	0.706	n.d.	0.052	n.d.
Q	22.3	11.46	n.d.	n.d.	0.013	0.013	0.155	n.d.	n.d.	n.d.	n.d.	—	n.d.	n.d.	0.23	n.d.	n.d.	n.d.	n.d.	n.d.	n.d.	n.d.
R	27.99	15.94	n.d.	0.023	n.d.	n.d.	n.d.	n.d.	0.058	0.013	n.d.	—	n.d.	n.d.	0.267	n.d.	n.d.	0.041	n.d.	n.d.	n.d.	n.d.
S	30.11	14.59	n.d.	0.023	n.d.	n.d.	n.d.	n.d.	n.d.	n.d.	n.d.	—	n.d.	n.d.	n.d.	n.d.	n.d.	n.d.	n.d.	0.027	n.d.	n.d.
T	26.71	28.88	0.319	0.022	n.d.	n.d.	0.058	n.d.	n.d.	0.008	n.d.	—	n.d.	n.d.	n.d.	n.d.	n.d.	0.382	n.d.	0	n.d.	n.d.

Mean	19.46	12.72	0.057	0.015	0.004	0.005	0.101	0.001	0.203	0.012	17.5	0.000	0.032	0.000	0.031	0.005	0.000	0.174	3.10	0.013	0.001	0.008
90° percentile	34.63	25.64	0.220	0.034	0.013	0.013	0.242	0.004	0.851	0.026	30.7	0.000	0.000	0.000	0.160	0.012	0.000	0.427	4.56	0.000	0.000	0.002
Standard deviation	10.44	12.46	0.109	0.029	0.010	0.012	0.129	0.004	0.550	0.028	51.7	0.000	0.106	0.000	0.080	0.015	0.000	0.512	13.8	0.084	0.007	0.031

## References

[1] Hernando MD, Mezcua M, Gómez MJ, Malato O, Agüera A, Fernández-Alba AR (2004). Comparative study of analytical methods involving gas chromatography-mass spectrometry after derivatization and gas chromatography-tandem mass spectrometry for the determination of selected endocrine disrupting compounds in wastewaters. *Journal of Chromatography A*.

[2] Castiglioni S, Bagnati R, Calamari D, Fanelli R, Zuccato E (2005). A multiresidue analytical method using solid-phase extraction and high-pressure liquid chromatography tandem mass spectrometry to measure pharmaceuticals of different therapeutic classes in urban wastewaters. *Journal of Chromatography A*.

[3] Castiglioni S, Fanelli R, Calamari D, Bagnati R, Zuccato E (2004). Methodological approaches for studying pharmaceuticals in the environment by comparing predicted and measured concentrations in River Po, Italy. *Regulatory Toxicology and Pharmacology*.

[4] Stumpf M, Ternes TA, Wilken RD, Rodrigues SV, Baumann W (1999). Polar drug residues in sewage and natural waters in the state of Rio de Janeiro, Brazil. *Science of the Total Environment*.

[5] Metcalfe CD, Koenig BG, Bennie DT, Servos M, Ternes TA, Hirsch R (2003). Occurrence of neutral and acidic drugs in the effluents of canadian sewage treatment plants. *Environmental Toxicology and Chemistry*.

[6] Johnson AC, Sumpter JP (2001). Removal of endocrine-disrupting chemicals in activated sludge treatment works. *Environmental Science and Technology*.

[7] Carballa M, Omil F, Lema JM (2004). Behavior of pharmaceuticals, cosmetics and hormones in a sewage treatment plant. *Water Research*.

[8] Ternes TA (1998). Occurrence of drugs in German sewage treatment plants and rivers. *Water Research*.

[9] Golet EM, Alder AC, Hartmann A, Temes TA, Giger W (2001). Trace determination of fluoroquinolone antibacterial agents in urban wastewater by solid-phase extraction and liquid chromatography with fluorescence detection. *Analytical Chemistry*.

[11] Hernando MD, Mezcua M, Fernández-Alba AR, Barceló D (2006). Environmental risk assessment of pharmaceutical residues in wastewater effluents, surface waters and sediments. *Talanta*.

[12] Beausse J (2004). Selected drugs in solid matrices: a review of environmental determination, occurrence and properties of principal substances. *Trends in Analytical Chemistry*.

[13] Thiele-Bruhn S (2003). Pharmaceutical antibiotic compounds in soils—a review. *Journal of Plant Nutrition and Soil Science*.

[14] Löffler D, Römbke J, Meller M, Ternes TA (2005). Environmental fate of pharmaceuticals in water/sediment systems. *Environmental Science and Technology*.

[15] Saccà ML, Accinelli C, Fick J, Lindberg R, Olsen B (2009). Environmental fate of the antiviral drug Tamiflu in two aquatic ecosystems. *Chemosphere*.

[16] Yang Y, Fu J, Peng H, Hou L, Liu M, Zhou JL (2011). Occurrence and phase distribution of selected pharmaceuticals in the Yangtze Estuary and its coastal zone. *Journal of Hazardous Materials*.

[17] Halling-Sørensen B, Nors Nielsen S, Lanzky PF, Ingerslev F, Holten Lützhøft HC, Jørgensen SE (1998). Occurrence, fate and effects of pharmaceutical substances in the environment—a review. *Chemosphere*.

[18] Díaz-Cruz MS, López de Alda MJ, Barceló D (2003). Environmental behavior and analysis of veterinary and human drugs in soils, sediments and sludge. *Trends in Analytical Chemistry*.

[19] Calamari D, Zuccato E, Castiglioni S, Bagnati R, Fanelli R (2003). Therapeutic drugs in the river Po and Lambro in Northern Italy: a strategic survey. *Environmental Science and Technology*.

[20] Hirsch R, Ternes T, Haberer K, Kratz K-L (1999). Occurrence of antibiotics in the aquatic environment. *Science of the Total Environment*.

[21] Tixier C, Singer HP, Oellers S, Müller SR (2003). Occurrence and fate of carbamazepine, clofibric acid, diclofenac, ibuprofen, ketoprofen, and naproxen in surface waters. *Environmental Science and Technology*.

[22] Weigel S, Kuhlmann J, Hühnerfuss H (2002). Drugs and personal care products as ubiquitous pollutants: occurrence and distribution of clofibric acid, caffeine and DEET in the North Sea. *Science of the Total Environment*.

[23] Bound JP, Voulvoulis N (2004). Pharmaceuticals in the aquatic environment: a comparison of risk assessment strategies. *Chemosphere*.

[24] EMEA (2006). Guidelines on the Environmental Risk Assessment of Medicinal Products for Human Use (Doc. Ref. EMEA/CHMP/SWP/447/00).

[25] O’ Brien E, Dietrich D (2004). Characterization of microcystin production in an Antarctic cyanobacterial mat community. *Trends in Biotechnology*.

[26] Wigley RL (1967). Comparative efficiencies of van Veen and Smith-Mclntyre grab samplers as revealed by motion pictures. *Ecology*.

[27] Gómez MJ, Petrović M, Fernández-Alba AR, Barceló D (2006). Determination of pharmaceuticals of various therapeutic classes by solid-phase extraction and liquid chromatography-tandem mass spectrometry analysis in hospital effluent wastewaters. *Journal of Chromatography A*.

[28] Dohrmann R (2006). Cation exchange capacity methodology I: an efficient model for the detection of incorrect cation exchange capacity and exchangeable cation results. *Applied Clay Science*.

[29] ISO Soil quality—determination of organic carbon by sulfochromic oxidation.

[30] Autorità di Bacino del fiume Po (2006). Caratteristiche del Bacino del fiume Po e primo esame dell’impatto ambientale delle attività umane sulle risorse idriche. *Monography*.

[31] Autorità di Bacino del fiume Po (2009). Atlante geomorfologico del fiume Po. *Monography*.

[32] Jjemba PK (2006). Excretion and ecotoxicity of pharmaceutical and personal care products in the environment. *Ecotoxicology and Environmental Safety*.

[33] Cooper ER, Siewicki TC, Phillips K (2008). Preliminary risk assessment database and risk ranking of pharmaceuticals in the environment. *Science of the Total Environment*.

[34] Zuccato E, Castiglioni S, Fanelli R (2005). Identification of the pharmaceuticals for human use contaminating the Italian aquatic environment. *Journal of Hazardous Materials*.

[35] Kuster M, López de Alda MJ, Hernando MD, Petrovic M, Martín-Alonso J, Barceló D (2008). Analysis and occurrence of pharmaceuticals, estrogens, progestogens and polar pesticides in sewage treatment plant effluents, river water and drinking water in the Llobregat river basin (Barcelona, Spain). *Journal of Hydrology*.

[36] Daughton CG, Ternes TA (1999). Pharmaceuticals and personal care products in the environment: agents of subtle change?. *Environmental Health Perspectives*.

[37] Pojana G, Fantinati A, Marcomini A (2011). Occurrence of environmentally relevant pharmaceuticals in Italian drinking water treatment plants. *International Journal of Environmental Analytical Chemistry*.

